# Antioxidant Supplementation on Sperm DNA Fragmentation and Sperm Parameters: A Systematic Review and Meta-Analysis

**DOI:** 10.5152/tud.2022.22058

**Published:** 2022-09-01

**Authors:** Bambang Sasongko Noegroho, Safendra Siregar, Kevin Anthony Glorius Tampubolon

**Affiliations:** 1 Department of Urology, Hasan Sadikin Academic Medical Center, Universitas Padjajaran Bandung, Indonesia

**Keywords:** Antioxidants, sperm DNA fragmentation, sperm parameters

## Abstract

**Objective:**

Infertility affects about 15% of couples in reproductive age worldwide. Male factors are the main causes for this condition. Antioxidants have long been used for infertility treatment as they are easily available with a low cost. In this systematic review, we focused on the relation between antioxidant supplementation and sperm DNA fragmentation and other sperm parameters.

**Material and methods:**

An online search from PubMed and ScienceDirect databases was conducted by 2 reviewers. We reviewed full-text articles to obtain detailed information.

**Results:**

Nine articles were included in this study. Four studies revealed a statistically significant reduction of sperm DNA fragmentation and five studies revealed an insignificant decrease of sperm DNA fragmentation. Sperm concentration, sperm motility, and morphology were also increased after antioxidant supplementation. Pregnancy rates were reported in 3 studies; the rates increased in 2 studies, and similar rate to placebo group was observed in 1 study.

**Conclusion:**

Antioxidant supplementation can counteract against oxidative stress and improve spermatogenesis process reflected by decrease of DNA Fragmentation Index (DFI), improvement of sperm parameters, and elevation of pregnancy rates confirmed by those included studies.

**Main Points**

- Infertility can be caused by poor sperm quality.
- Imbalance of free radicals and antioxidants in sperm leads to DNA fragmentation.
- Antioxidant supplementation can counteract against oxidative stress and improve spermatogenesis.

## Introduction

Infertility is a global health problem, defined as the inability to get pregnant after 1 year of unprotected sexual intercourse.^1^ It involves approximately 15% of couples worldwide, and male factors account for 40%-50% of the cases.^2,3^

Antioxidants are widely available at low cost, and hence treatment with antioxidants has more benefits than other fertility treatments. They play an important role in impeding the oxidation of molecules through scavenging free radicals directly or chelation of redox metals.^2^ They form the primary defense as they inhibit the reactive oxygen species (ROS) formation by binding ions and enzymatic antioxidants, which regulate the gene expression of oxidative enzymes. Antioxidants can be supplemented orally as either a single component or mixed components.^3^ The most common antioxidants obtained from dietary supplements, which are often studied for their correlation with male subfertility, are vitamin C, vitamin E, carotenoids, carnitines, ubiquinol, cysteine, folate, selenium, and zinc.^4^

Imbalance of free radicals and antioxidants in sperm leads to DNA fragmentation (DNA strand breaks). Free radicals in semen are usually synthesized by leukocytes and spermatozoa, enzymes such as superoxide dismutase, catalase, and glutathione peroxidase, and nonenzymes such as ascorbic acid, tocopherol, glutathione, albumin, carnitines, carotenoids, flavonoids, urate, and prostasomes.^5^

Sperm DNA fragmentation (SDF) may also be the consequence of the weak anatomical structure of the sperm chromatin, increasing the susceptibility of DNA damage due to oxidative stress (OS). Major causes of SDF include abnormal sperm synthesis and chromatin remodeling fault. It actually has a wide definition that consists of: (1) single- or double-DNA strand breaks, (2) base deletion or modification, (3) interstrand or intrastrand cross-linkage, and (4) DNA–protein cross-linkage.^6,7^ Sperm DNA fragmentation >30% has been correlated with delayed pregnancy and reflect as one of the factors of pregnancy failure.^6,7^

Antioxidant oral supplementation can improve the quality of sperm as it may lower the OS. Therefore, antioxidant supplements have been studied more recently even though the optimal evidence for antioxidant dose for clinical use remains undetermined yet. In this systematic review, we focused on the relation between antioxidant supplementation and SDF and other sperm parameters.

## Materials and Methods

### Eligibility Criteria

Articles were included if they met the following criteria:

1. English and full-text articles were available.
2. Articles were published between January 2016 and January 2022.
3. The studies were randomized controlled trials or cohort studies.
4. The studies reported DFI and/or sperm parameters pre- and post-antioxidant supplementation among infertile patients.

### Guidelines

The guidelines by Preferred Reporting Items for Systematic Reviews and Meta-analysis (PRISMA) were used for reporting this study.^8^

### Search Strategy

An online literature search was performed on PubMed, ScienceDirect, Scopus, and Embase databases following PRISMA guidelines. The search was conducted on January 2, 2022, using the search term (“antioxidants supplements”) AND (“dna fragmentation index” OR “sperm dna fragmentation”) AND (“sperm parameters” OR “sperm analysis” OR “semen parameter” OR “semen analysis”). Since this systematic review did not involve human investigation, ethics approval was not required.

### Data Extraction and Quality Assessment

Literatures were selected by two reviewers, and data were extracted to an Excel database. Both reviewers screened for titles and abstracts to determine the eligible articles. Then, we did a full-text review to obtain detailed information.

### Risk of Bias Assessment

**Figure 1.**
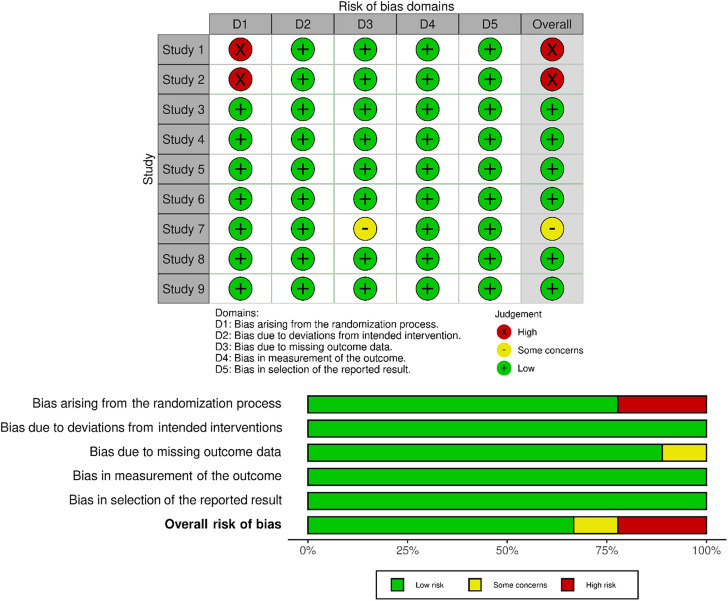
Risk of bias assessment.

A total of nine articles met the inclusion criteria and were analyzed for potential bias according to Cochrane Risk of Bias Assessment Tools (Figure 1). Most of the included studies were at low risk of bias, and we excluded those studies which presented bias in more than one of these categories. We found that 2 articles were at high risk of bias due to the selection design as non-randomized trials. A study had some concerns in the risk of bias with regard to missing outcome data of 2 patients who missed to be present during the visit time.

## Results

### Study Selection

**Figure 2.**
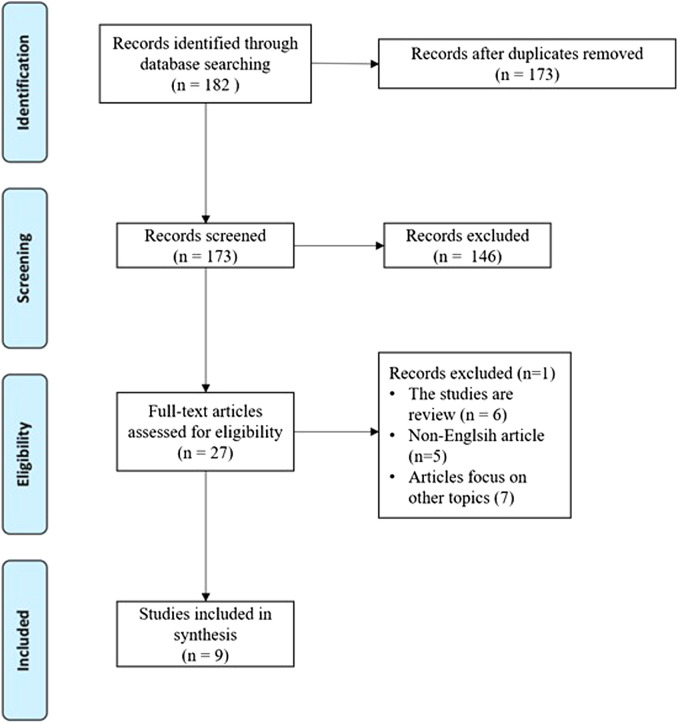
Preferred reporting items for systematic reviews and meta-analysis flowchart.

An online search from all databases resulted in 182 articles. Duplicates were checked and excluded, leaving 173 articles. A total of 27 articles were found to be relevant to this study. After a full-text review, nine articles were included for this review (Figure 2).

### Characteristics of the Included Studies

Nine studies fulfilled the inclusion criteria and were selected for the systematic review. Among the studies, 3 studies were prospective cohort studies and six randomized clinical trials. Terminal deoxynucleotidyl transferase dUTP nick end labeling assay was used in 3 studies, sperm chromatin structure analysis (sperm chromatin structure analysis in three studies, and sperm chromatin dispersion in 2 studies, while 1 did not specify the assay. The duration of antioxidant supplementation was 3-6 months. The results of our study are presented in Tables 1 and 2.

Antioxidant supplements used in those studies varied widely and included docosahexaenoic acid (DHA), *N*-acetyl-cysteine (NAC), l-carnitine, acetyl-l-carnitine, multivitamins, coenzyme Q10, omega-3, oligoelements, and other micronutrients.

### Sperm DNA fragmentation

**Figure 3.**
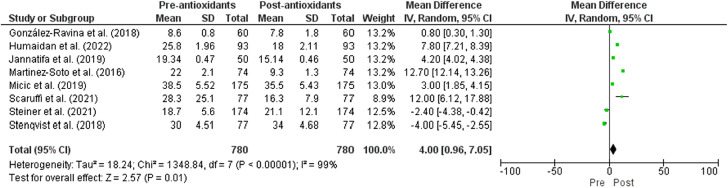
A forest plot of sperm concentration post-antioxidant supplementation.

Over of these Nine studies showed that SDF percentage positively decreased post-antioxidant supplementation. Four studies revealed a statistically significant reduction of SDF. In the study by Martinez-Soto et al.^9^ a reduction of SDF% from 22.0% ± 2.1% to 9.3% ± 1.3% was observed (*P* < .01) post 1500 mg DHA daily administration. Sperm DNA fragmentation also decreased from 25.8% to 18.0% after oral antioxidant treatment (*P* < .001) that consisted of multivitamins, coenzyme Q10, omega-3, and oligoelements in a study by Humaidan et al.^10^ after the administration of NAC, the patients showed lower SDF (18%) when compared to that before the supplement administration (25.8%).^11^ In the Stenqvist et al’s study, the following antioxidant components were used: l-carnitine 750 mg, coenzyme Q10 10 mg, and folic acid 100 µg, resulting in a reduction of SDF% (pre 34% vs post 30%) (*P* > .01) within 6 months. Micic et al^12^ revealed a decrease of SDF% (pre 38.5% (32.00-48.70%) vs. post 35.50% (25.50-44.00%)) (*P* < .001) after the administration of Proxeed Plus. A multicenter longitudinal, prospective study revealed that the administration of myo-inositol, alpha-lipoic acid, folic acid, coenzyme Q10, zinc, and selenium and vitamins B2, B6, and B12 showed a reduction of SDF% (pre 28.3% ± 25.1% vs. post 16.3% ± 7.9%) (*P* = .078).^5^ A forest plot showed that sperm concentrations post-antioxidant supplementation were significantly higher than those of pre-antioxidant supplementation (mean difference 4.00; 95% CI, 0.96-7.05; *P* = .01) (Figure 3).

### Sperm Concentration

**Figure 4.**
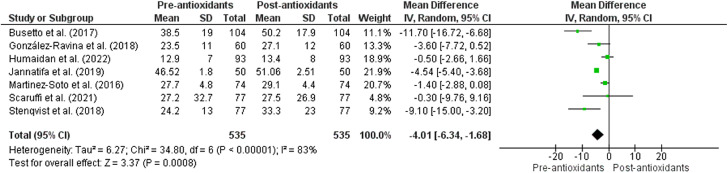
A forest plot showing sperm concentration pre-antioxidant supplementation.

Nine studies revealed that sperm concentration was elevated after antioxidant supplementation. Significant *P* values were observed in 3 studies as they increased from 27.7% ± 4.8% to 29.1% ± 4.4% (*P* < .01),^9^ 46.5% ± 1.80% to 51.0% ± 2.51% (*P* < .02),^11^ and 27.2% ± 32% to 27.5% ± 26.9% (*P* = .027),^5^ respectively, and other 6 studies showed statistically insignificant increase of sperm concentration. A forest plot showed that sperm concentration pre-antioxidant supplementation was significantly lower than post-antioxidant supplementation (mean difference −4.01; 95% CI, −6.34 to −to 34d*P* < .001) (Figure 4).

### Sperm Motility

**Figure 5.**
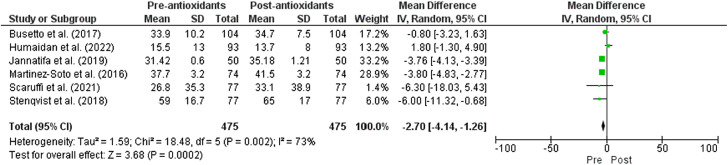
A forest plot showing total sperm motility.

**Figure 6.**
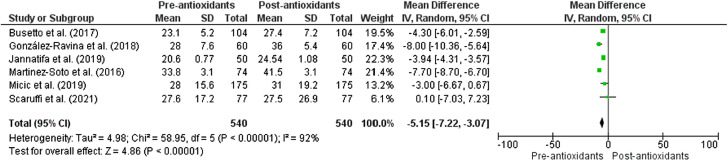
A forest plot showing progressive sperm motility.

Increment total and progressive sperm motility also denoted post-treatment of antioxidants (mean difference −5.15; 95% CI, −7.22 to −3.07; *P* < .001) (Figures 5 and 6). Total sperm motility was significantly higher in 3 studies post-treatment compared to pre-treatment with antioxidants (mean difference −2.70; 95% CI, −4.14 to −1.26; *P* < .001).

### Sperm morphology

**Figure 7.**
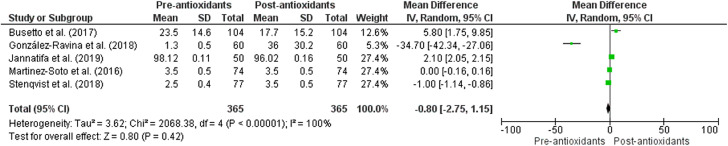
A forest plot showing sperm morphology.

Sperm morphology was found to vary after antioxidant supplementation. Mostly, it tends to be elevated after antioxidant supplementation, while several studies revealed a decrease in or similar sperm morphology before and after treatment with antioxidants .^12,13^ A meta-analysis showed that sperm morphology before antioxidant supplementation was insignificantly lower than that after antioxidant supplementation (mean difference −0.80; 95% CI, −2.75 to 1.15; *P* = .42) (Figure 7).

### Pregnancy rate

We found that 3 studies represented the pregnancy rates among those patients. One study found that 10 pregnancies occurred during the follow-up time within the post-supplemented group and another study found three pregnancies within the supplemented group, among which 1 was through In vitro fertilization (IVF) and 2 were spontaneous pregnancies. A multicenter, double-blind, randomized, placebo-controlled trial study found similar pregnancy rates between the antioxidant-supplemented group and the placebo group.

## Discussion

Infertility was reported in approximately 15% of couples in reproductive age worldwide, and male factors were referred as the predominant causes.^2,3^ There are multiple factors that affect infertility, which include genetic disorders, dietary habits, lifestyle, and environment factors.^14^ Etiopathogenesis of male infertility involved diverse aspects, and OS is one of them. The main component of free radicals that lead to decreased spermatogenesis is ROS.^1^

Antioxidants (e.g., vitamin C, vitamin E, glutathione, albumin, carotenoids, or uric acid) were plentiful in the seminal plasma. They act to avoid sperm fragmentation caused by ROS following ejaculation. When OS occurred, these antioxidants would be inadequate. Imbalance of free radicals and antioxidant levels leads to OS event which reduces sperm quality. Therefore, sufficient antioxidants should be available to resist against the abundance level of free radicals such as ROS.^14^

Antioxidant scavenging structures have a critical part to play in the process to inactivate ROS. Various antioxidant supplements and combinations of regimens, such as vitamins C and E, selenium, zinc, and glutathione, have long been used as treatments for male infertility.^1^ The correlation between SDF and semen ROS is the fundamental value of antioxidant consumption. It is purposed to improve sperm quality.^1,14,15^

Scaruffi et al^5^ evaluated reproductive outcomes of IVF cycles after treatment with 2 Gametogen® tablets that contained myo-inositol (1000 mg), alpha-lipoic acid (800 mg), folic acid (400 mg), coenzyme Q10 (200 mg), zinc (15 mg), and selenium (83 µg) and vitamins B2 (2.8 mg), B6 (2.8 mg), and B12 (5 µg).^5^ The study exhibited significant progressive sperm motility and pregnancy rate (*P* < .001).^5^ Pregnancy rate increased from 3% pre-treatment to 33% 12 weeks post-treatment.^5^

### Docosahexaenoic Acid

In the present study, we found that 4 articles revealed a significant decrease of SDF after various antioxidant supplementations. González-Ravina et al^14^ and Martinez-Soto et al^9^ used DHA to evaluate the antioxidant effects against SDF and sperm parameters. They showed improvement of sperm parameters and DFI reduction.^9^

Docosahexaenoic acid is the one of the sperm lipid membrane components which hydrates the plasma membrane to allow fusion-related fertilization process. This supplementation would give advantages to sperm integrity and quality through the mechanism of elevated intrinsic antioxidant synthesis (glutathione or catalase), maintaining the integrity of DNA, and synthesizing anti-inflammatory mediators.^16^ Several studies have analyzed the effects of dietary omega-3 polyunsaturated fatty acids (PUFA) supplementation on sperm quality in different species of domestic animals and humans.^17^

Safarinejad et al^17^ evaluated the effects of omega-3 fatty acid (a concentration of 1840 mg/day, i.e., 720 mg of DHA and 1120 mg of eicosapentaenoic (EPA)) supplementation for 32 weeks.^17^ The study revealed a significant improvement of sperm motility, concentration, normal morphology, and antioxidant status.^17^ Furthermore, omega-3 PUFA levels (DHA and EPA) were increased in spermatozoa and in seminal plasma.^17^ A study by Humaidan et al^10^ that used omega-3 as oral antioxidants showed good outcomes of SDF, sperm concentration, and total sperm motility.^10^

### *N*-acetyl-cysteine

A randomized, blinded clinical trial study that used NAC 600 mg/day for 3 months showed significant SDF% reduction and sperm parameters.^11^ It is an amino acid derivative of l-cysteine, which plays an antioxidant role. *N*-acetyl-cysteine, the same way as DHA, also supports glutathione production.^11^

### l-Carnitine and Acetyl-l-Carnitine

Studies by Busetto et al^16^ and Micic et al^12^ used Proxeed Plus supplements that consisted of 1000 mg l-carnitine, 725 mg fumarate, 500 mg acetyl-l-carnitine, 1000 mg fructose, 20 mg coenzyme Q10, 90 mg vitamin C, 10 mg zinc, 200 μg folic acid, and 1.5 μg vitamin B12 for 6 months. They showed an increase of sperm motility and morphology and a decrease of SDF that were statistically significant. l-Carnitine and acetyl-l-carnitine are known to play a key part in spermatozoa energy metabolism. Clinical studies have previously revealed that oral administration of these compounds to asthenozoospermic individuals increases the percentage of total sperm motility, progressive sperm motility, average speed, and linearity of sperm motility.^18,19^

Stenqvist et al administered combination of vitamins, antioxidants consisting of l-carnitine 750 mg, coenzyme Q10 10 mg, and folic acid 100 µg or mcg (microgram), and oligoelements (zinc 5 mg and selenium 25 mg) which also showed similar results as aforementioned studies except for increase of total sperm motility. Pregnancies were also reported in this study in 3 patients who were given antioxidants and in 4 patients in placebo group.^15^ The association between pregnancy and DFI levels is known. Low DFI levels (<30%) can decrease the spontaneous pregnancy occurrence.^20^

A study that involved 264 couples who were given supplements that comprised of 500 mg vitamin C, 400 mg vitamin E, 0.20 mg selenium, 1000 mg l-carnitine, 20 mg zinc, 1000 mcg folic acid, and 10 mg lycopene daily for 3 months showed insignificant results of DFI and sperm parameters.^13^ One limitation of this study is that not all antioxidant types were involved in this study. We suggest finding a correlation with another antioxidant for the future. In conclusion, this study discovers that antioxidant supplementation can counteract against OS and improve spermatogenesis, reflected by a decrease of DFI, an improvement of sperm parameters, and an elevation of pregnancy rates among those patients. This therapy can be an option for infertility treatment when used either in combination with other treatments or as a single therapy.

## Figures and Tables

**Table 1.** Study Characteristics

No.	Authors (Year)	Antioxidant Supplements	Type of Study	Number of Participants	Effective Dose	Duration	SDF Assay	Main SDF Results
1	Humaidan et al^10^	Oral antioxidant therapy (multivitamins, coenzyme Q10, omega-3, and oligoelements)	Prospective study	93	Coenzyme Q10 100 mg (OmniQ10 Energy, Biosym A/S), one multivitamin (Apovit®) tablet, and 1 g omega-3 (Apovit®) daily	3 months	SCSA	Reduction of SDF% (pre 25.8% vs. post 18.0%) (*P* < .001)
2	Scaruffi et al^5^	Myo-inositol, alpha-lipoic acid, folic acid, coenzyme Q10, zinc, and selenium and vitamins B2, B6, and B12	Multicenter, longitudinal, prospective study	77	Myo-inositol (1000 mg), alpha-lipoic acid (800 mg), folic acid (400 mg), coenzyme Q10 (200 mg), zinc (15 mg), and selenium (83 µg) and vitamins B2 (2.8 mg), B6 (2.8 mg), and B12 (5 µg)	12 weeks	SCD	Reduction of SDF% (pre 28.3% ± 25.1% vs. post 16.3% ± 7.9% (*P* = .078)
3	Steiner et al^13^	Vitamin C, vitamin E, selenium, l-carnitine, zinc, folic acid, and lycopene	Multicenter, double-blind, randomized, placebo-controlled trial with an internal pilot study	174	500 mg vitamin C, 400 mg vitamin E, 0.20 mg selenium, 1000 mg l-carnitine, 20 mg zinc, 1000 mcg folic acid, and 10 mg lycopene daily	3 months	SCSA	Treatment group (18.7) vs. placebo group (21.1)
4	Jannatifa et al^11^	NAC	Randomized, blinded clinical trial	50	NAC 600 mg/day by oral route for 3 months	3 months	TUNEL assay	Reduction of SDF% (pre 19.34% ± 0.47% vs. post 15.14% ± 0.46%) (*P* < .001)
5	Micic et al^12^	Proxeed Plus (l-carnitine, acetyl- l -carnitine, and other micronutrients)	Prospective, randomized, double‐blind, placebo‐controlled clinical trial	175	1000 mg l-carnitine, 725 mg fumarate, 500 mg acetyl-l- carnitine, 1000 mg fructose, 20 mg coenzyme Q10, 90 mg vitamin C, 10 mg zinc, 200 μg folic acid, and 1.5 μg vitamin B12	6 months	SCD	Reduction of SDF% (pre 38.5% (32.00-48.70%) vs. post 35.50% (25.50-44.00%) (*P* < .001)
6	González-Ravina et al^14^	DHA	Prospective, randomized, double-blind, placebo-controlled study	60	1 g	3 months	TUNEL	Reduction of SDF% (pre 8.6% vs. post 7.8%) (*P* = .7)
7	Stenqvist et al^15^	Vitamins, antioxidants, oligoelements, maltodextrin, calcium carbonate, citric acid, steviol glycoside, flavors, beta-carotene, and silicon dioxide	Placebo-controlled, double-blind, randomized study	77	Vitamin C 30 mg, vitamin E 5 mg, vitamin B12 0.5 μg, l-carnitine 750 mg, coenzyme Q10 10 mg, folic acid 100 ug	6 months	SCSA	Reduction of SDF% (pre 30% vs. post 34%) (*P* > .01)
8	Busetto et al^16^	l-carnitine, acetyl-l-carnitine, and other micronutrients	Monocentric, randomized, double-blind, placebo-controlled trial	104	Proxeed Plus consisted of 1000 mg l-carnitine, 725 mg fumarate, 500 mg acetyl-l-carnitine, 1000 mg fructose, 20 mg coenzyme Q10, 90 mg vitamin C, 10 mg zinc, 200 μg folic acid, and 1.5 μg vitamin B12	3 months	N/A	N/A
9	Martinez-Soto et al^9^	DHA	Randomized, double-blind, placebo-controlled, parallel group study	74	1500 mg oil per day	6 months	TUNEL	Reduction of SDF% (pre 22.0% ± 2.1% vs. post 9.3% ± 1.3%) (*P* < .01)

NAC, *N*-acetyl-cysteine; SCD, sperm chromatin dispersion; SCSA, sperm chromatin structure analysis; SDF, sperm DNA fragmentation; TUNEL, terminal deoxynucleotidyl transferase dUTP nick end labeling.

**Table 2.** Sperm Parameters

No.	Authors (Year)	Sperm Concentration (10^6^ Cells/mL)	Total Sperm Motility (%)	Progressive Sperm Motility (%)	Sperm Morphology (%)	Pregnancy Rate
1	Humaidan et al^10^	Pre 12.9% vs. post 13.4%	Pre 15.5% vs. post 13.7%	N/A	N/A	N/A
2	Scaruffi et al^5^	Pre 27.2% ± 32.7% vs. post 27.5% ± 26.9% (*P* = .027)	Pre 26.8% ± 35.3% vs. post 33.1% ± 38.9% (*P* = .003)	Pre 27.6% ± 17.2% vs. post 27.5% ± 26.9% (*P* = .027)	N/A	N/A
3	Steiner et al^13^	Treatment group 21.0% vs. placebo group 16.7%	Treatment group 44.9% ± 17.3% vs. placebo group 43.0% ± 15.7%	N/A	Treatment group 4.0% vs. placebo group 6.0%	Treatment group 9% vs. placebo group 9%
4	Jannatifa et al^11^	Pre 46.52% ± 1.80% vs. post 51.06% ± 2.51% (*P* < .02)	Pre 31.42% ± 0.60% vs. post 35.18% ± 1.21% (*P* < .01)	Pre 20.60% ± 0.77% vs. post 24.54% ± 1.08% (*P* < .001)	Pre 98.12% ± 0.11% vs. post 96.02% ± 0. 16% (*P* < .001)	N/A
5	Micic et al^12^	N/A	N/A	Pre 28.0% (12.0-38.0%) vs. post 31.0% (20.0-41.0%) (*P* < .001)	N/A	N/A
6	González-Ravina et al^14^	Pre 23.5% to post 27.1% (*P* = .73)	N/A	Pre 28% to post 36% (*P* = .002)	Pre 1.3% to post 3.6% (*P* < .001)	N/A
7	Stenqvist et al^15^	Pre 24.2% to post 33.3% (*P* > .01)	Pre 59.0% to 65.0% (*P* > .01)	N/A	Pre 2.5% to post 3.5% (*P* < .001)	Three pregnancies occurred in the supplementation group (one IVF and two spontaneous pregnancies)
8	Busetto et al^16^	Pre 38.5% ± 19.0% to 50.2% ± 17.9% (*P* = .04)	Pre 33.9% ± 10.2% vs. post 34.7% ± 7.5% (*P* = .005)	Pre 23.1% ± 5.2% vs. post 27.4% ± 7.2% (*P* = .01)	Pre 23.5% ± 14.6% vs. post 17.7% ± 15.2% (*P* = .005)	Ten pregnancies occurred in the supplementation group
9	Martinez-Soto et al^9^	Increase of concentration (pre 27.7% ± 4.8% vs. post 29.1% ± 4.4%) (*P* < .01)	Increase of total motility (pre 37.7% ± 3.2% vs. post 41.5% ± 3.2%)	Increase of total motility (pre 33.8% ± 3.1% vs. post 41.5% ± 3.1)	Pre 3.5% ± 0.5% vs. post 3.5% ± 0.5%	N/A
